# Effectiveness of the ‘Weet wat je eet’ nutrition education programme in Dutch secondary schools

**DOI:** 10.1017/jns.2024.80

**Published:** 2024-12-20

**Authors:** Femke A. Hoefnagels, Simone Versteegt, Annette Stafleu, Marthe C. Huigens, Marieke C. E. Battjes-Fries

**Affiliations:** 1 Department of Nutrition and Health, Louis Bolk Institute, Bunnik, The Netherlands; 2 Independent Researcher; 3 The Netherlands Nutrition Centre (het Voedingscentrum), The Hague, The Netherlands

**Keywords:** Adolescence, Effectiveness, Nutrition education, Secondary schools, Students, COM-B model, Capability, Opportunity, Motivation and Behaviour model, GDPR, General Data Protection Regulation, IQR, Interquartile Range, SD, Standard Deviation, WHO, World Health Organization, WWJE, ‘Weet wat je eet’

## Abstract

To assess the effectiveness of the ‘Weet wat je eet’ (‘Know what you eat’) school-based nutrition education programme on behavioural determinants and behaviour among students aged 12–15 years. A quasi-experimental study design was used, collecting data at baseline and after implementing the programme in both an intervention and control group (in total 611 students) across the Netherlands. Students from eighteen Dutch secondary education schools completed two consecutive questionnaires, assessing knowledge, self-efficacy, attitude, subjective norm, intention, and behaviours related to healthy, safe, and sustainable nutrition. Multilevel regression analyses were conducted corrected for gender, grade, education level, and school location. The intervention group showed a significant higher increase in self-efficacy, attitude, intention to drink water (all three *P < 0.01)*, and a significant higher decrease in the consumption of sugary drinks, snacks, and meat (all *P < 0.05*) than the control group. Both the groups scored significantly higher on knowledge during the post-test (both *P < 0.05),* although the intervention group not significantly higher than the control group (P = 0.14). No significant effects were observed for subjective norm, intention, and fruit, vegetable, and whole grain bread consumption. The results of this study showed positive effects of the ‘Weet wat je eet’ school-based nutrition education programme on self-efficacy and attitude towards healthy, safe and sustainable nutrition, intention to drink more water, and various healthy eating behaviours among secondary school students. Further research is necessary to assess the long-term sustainability of these results.

## Introduction

For the promotion of good health and a sustainable environment, it is crucial for young people to adopt a healthy, safe, and sustainable eating pattern.^(1,2)^ Optimal growth, development, and health during childhood and adolescence necessitate a healthy dietary pattern.^(3)^ Research has demonstrated that a healthy diet enhances children’s cognitive skills such as concentration and memory, improves mood and energy levels, and boosts academic performance.^(4)^ Moreover, a healthy eating pattern can reduce the burden of non-communicable diseases.^(3,5)^ Besides, shifting towards a more plant-based and less animal-based eating pattern is not only beneficial for health but also for the environment.^(6)^ Raising awareness about sustainability and food safety, including aspects like food origin, food waste, and food hygiene, is vital for imparting knowledge to young people, enabling them to make healthy, safe, and sustainable food choices in the future. Nutrition behaviour established during childhood often persist into later stages of life.^(7)^ Adolescence is an important period of mental, physical, and social development. Nutrition behaviour, as well as other health-related habits, developed during adolescence tend to persist into later stages of life, making the teenage years an important time to encourage healthy and sustainable eating behaviours.^(7,8)^

Schools provide an ideal environment for educating children and adolescents about healthy, safe, and sustainable nutrition due to their existing infrastructure. Students from diverse backgrounds spend a significant amount of time in schools each week, acquiring knowledge, skills, and adopting favourable behaviours can be of benefit later in life.^(9,10)^ The school environment has been identified by the WHO as an ideal setting in which youth consume approximately one-third to one-half of their daily food intake.^(11)^ In the Netherlands, the proportion of the daily food intake consumed at school compared to total food consumption in grams is, with approximately 15%, lower.^(12)^ Contento defined nutrition education as ‘any combination of educational strategies, accompanied by environmental supports, designed to facilitate voluntary adoption of food choices and other food and nutrition-related behaviours conducive to health and well-being’.^(13)^ For primary education, numerous nutrition education programmes are available, of which the effectiveness have been proven.^(13–16)^ Presently, there exists limited empirical evidence regarding the efficacy of nutritional education interventions targeting adolescents in general and within the context of Dutch secondary education.^(17,18)^ However, little is known yet about the effectiveness of nutrition education among adolescents in Dutch secondary schools.

‘Weet wat je eet’ (it could be translated as ‘Know what you eat’, but this doesn’t capture the wordplay in the original Dutch title) is a school-based nutrition education programme with the objective to empower young people to make healthy, safe, and sustainable food choices and foster a positive attitude towards these choices. The programme is developed by the Netherlands Nutrition Centre, aimed at the lower grades of secondary school education and secondary vocational education. In the Netherlands, secondary education is divided in different levels: university preparatory education, senior general secondary education and preparatory secondary vocational education. It is an online programme, consisting of six lessons that teach students about healthy, safe, and sustainable nutrition. The lessons cover various key topics, for example, the functions of different nutrients, making informed choices based on food labels, the importance of consuming food groups such as vegetables, and safe food handling and preparation practices to prevent foodborne illnesses. Additionally, the programme addresses the environmental impact of food choices, reducing food waste and understanding the environmental footprint of different foods. The intervention is delivered through a digital learning environment where students can engage independently with the material at their own pace on their own devices, such as a laptops, iPads, or smartphones. The digital learning environment includes interactive modules, videos, and quizzes. Practical assignments, such as meal planning and cooking exercises, allow students to apply theoretical knowledge in real-life scenarios. Personalised feedback is provided based on students’ responses in the quizzes, addressing their questions and tailoring the programme to their needs. According to the Netherlands Nutrition Centre, more than 15,000 students followed the recently revised programme in 2022, which is freely available at wwje.nl.^(19)^

The focus of this study is to assess the effectiveness of the ‘Weet wat je eet’ programme of the Netherlands Nutrition Centre, specifically targeting students in all levels of secondary education in the lower grades and thereby contributing to insights in the effectiveness of nutrition education for adolescents in secondary education in general.

## Methods

### Study design

In a quasi-experimental study design, data on behavioural determinants and behaviour towards healthy, safe, and sustainable nutrition were collected, including a pre-test and post-test in an intervention group and control group. The pre- and post-test in the intervention group were conducted using digital questionnaires among secondary school students at baseline and after implementing the lessons from the programme in the autumn of 2022. This intervention group was compared to a control group consisting of similar secondary schools who also completed a digital questionnaire during both a pre-test and a post-test, but that did not implement the lessons during the same period.

### Participants

Schools that had previously implemented the programme were contacted to participate in the study by the Netherlands Nutrition Centre. Additionally, schools were recruited through recruitment texts via LinkedIn, various newsletters, and within the digital ‘Weet wat je eet’ environment. Schools were also informed about this study at the conference of the Netherlands Institute for Biology. Interested teachers had the opportunity to sign up for participation by completing a contact form.

The following inclusion criteria were applied for participation in the study: students from the first and second grades of all levels of secondary education, who had not previously followed the programme. Prior to participating in the study, students were asked to provide informed consent. The goal was to recruit approximately 400 students from ten schools per condition (intervention or control group), based on the power calculation for the effect studies on the ‘Taste Lessons’ educational programme in primary education on behavioural determinants.^(20)^ Out of the 23 schools that initially expressed interest in participating, a total of 18 schools actively took part in the study. Seven schools chose to implement the programme, while 11 were part of the control group. The remaining five schools withdrew from participation due to not meeting the inclusion criteria, for example, different school type.

### Procedures

The teachers from participating schools received two personalised links to the digital questionnaires, one for the pre-test and one for the post-test, intended for all students in their class(es) to be filled out during school time. These links were distributed to the students using the teachers’ preferred method, such as sharing them on the student portal or any other convenient means. The pre-test questionnaire was administered to both the intervention and control group before the students in the intervention group started with the first lesson of the programme, between September and November 2022. The post-test questionnaire was administered to the intervention group after they completed the lessons and to the control group during the same period, within a timeframe of three to seven weeks, from November 2022 to January 2023. Participants spent an average of 9 minutes completing the questionnaire. The students engaged in an average of 5.0 lessons out of the 6 lessons offered in the educational programme (SD: 1.60).

Participation in the research and the implementation of the programme were both voluntary. Students were not obliged to complete the questionnaires if they chose not to, they had the freedom to withdraw from the study at any time without having to explain why. All data collected were treated as confidential and processed in a coded manner to ensure anonymity and privacy. This study was conducted according to the guidelines laid down in the Declaration of Helsinki. The medical ethical review committee of VU Amsterdam University exempted this study from ethical approval (reference: 2022.0305). As a token of appreciation for their participation, all classes that completed both questionnaires received a classroom flowerpot and they had the chance to win a food truck for one day.

### Outcome measures

The outcome measures were selected and the questionnaires were designed based on the COM-B model. The COM-B model demonstrates that human behaviour (B) results from the interaction between personal physical and psychological capabilities (C), to utilise social and environmental opportunities (O) via motivators (M) that are reflective (thinking with the head) or automatic (emotional-‘thinking’ with the heart).^(21)^ In the questionnaire, each component of the COM-B model was aligned with the intervention. In the context of the intervention, capabilities were operationalised as knowledge and self-efficacy. For knowledge this included for example nutritional basics, food label interpretation, and safe food handling. Self-efficacy was assessed through questions on students’ understanding and confidence in making informed food choices. Opportunities were operationalised as subjective norms through questions about students’ perceptions of their social groups. Motivation was operationalised via attitudes and intentions, by assessing students’ attitudes towards healthy, safe, and sustainable eating, and their intentions to adopt specific behaviours covered in the lesson content. The operationalisation of the COM-B model is also been used in other studies.^(22)^ The interplay between these components is crucial for behaviour. By enhancing capabilities, providing opportunities and influencing motivators, the model creates a comprehensive approach to measure behaviour. The target behaviour was operationalised as several healthy, safe, and sustainable food behaviours, also as covered in the lesson content. The physical capabilities, for example, age and attendance, and environmental opportunities, for example, availability of the educational programme, were fixed and thus outside the scope of this research. The COM-B model has been successfully applied to understand children’s health behaviours within the domains of medical and education science,^(23)^ as well as in designing and evaluating interventions aimed at improving children’s physical activity levels.^(24)^

To assess all outcome measures and the respondents’ demographic characteristics, four to nine items per determinant and one to three questions per topic (healthy, safe, and sustainable) were included in the questionnaire (Table 1 for example questions). For the intervention group in the post-test questionnaire, supplementary questions were included to gather feedback on the programme, with an overall evaluation on a scale of 1–10 and specific parts of the programme on a scale ranging from low (=1) to high (=5). In the questions the same terminology was used as in the lessons, to ensure consistency. Furthermore, the questions’ readability was examined against B1-level standard and discussed with teachers. To ensure usability and comprehensibility, a group of young individuals tested the questionnaire and subsequently the questions were adapted accordingly.

**Table 1.** Content of the questionnaires, answer scales and number of items

Determinant	Content of the questions	Example questions	Answer scale	Items
Demographic characteristics	Name, age, gender, grade, and education level	What is your gender?	Different answer options	5
**Capacity**				
Knowledge	Questions about healthy, safe, and sustainable nutrition	The temperature inside the fridge varies. Where in the refrigerator is it recommended to store dairy items such as milk, yogurt, and cream?	Multiple-choice with 4 options	9
Self-efficacy	I can…	I can provide examples of how influencers promote (un)healthy products.	Disagree (=1) to agree (=5)	6
**Opportunity**				
Subjective norm	My friends consider … to be …	My friends consider animal welfare to be …	Unimportant (=1) to important (=7)	4
**Motivation**				
Attitude	I consider … to be …	I consider healthy eating to be …	Unimportant (=1) to important (=7)	4
Intention	I plan to … in the upcoming month	I intend to drink more water in the upcoming month.	Disagree (=1) to agree (=5)	7
**Behaviour**				
Behaviour	I generally eat/drink…	I generally drink sugary beverages *(like cola, orange soda, and fruit juices)*	Times per week, per day or how often	6

### Statistical analyses

IBM SPSS version 26 was used for the analyses. Normally distributed continuous variables are reported as mean ± standard deviation while categorical variables are presented as n (number) and percentage. Non-normally distributed continuous variables are presented as median and interquartile range.

Responses of the pre- and post-test questionnaires were linked to each other based on the first name and class of the students. Out of the initial 1,072 responses on the pre-test questionnaire and 946 from the post-test questionnaire across eighteen participating schools, 611 students (57% of the pre-test responses) were successfully linked based on their first name and class, and these students answered at least all knowledge questions, which constituted the first substantive questions in the questionnaire. Of the students who answered all knowledge questions, 96% also answered the last question of the questionnaire.

The average scores for all determinants were calculated considering questions within a determinant only if their Cronbach’s alpha was at least 0.7. The Cronbach’s alpha for the determinants ranged from 0.71 to 0.84, indicating satisfactory internal consistency. The difficulty level of the knowledge questions was assessed using the facility index. Questions in the pre-test that were answered correctly by more than 80% of the students (considered too easy) or less than 20% of the students (considered too difficult) were excluded from further analyses.^(25)^ One knowledge question had a correct response rate exceeding 80% in the pre-test (specifically, the question ‘what is a healthy food switch?’ with 89% correct answers), and therefore, this question was excluded in the subsequent analyses.

Paired *t*-tests were used to compare pre- and post-test scores separately for the intervention and control groups. Independent *t*-tests were employed to assess differences between the control and intervention group at each measurement point. The change in the average score for each determinant was calculated by subtracting the pre-test score from the post-test score for each student. These change scores were used as outcome measures in the further analyses.

To account for the clustering effect of students within the same school, multilevel linear regression analyses were conducted. Within these analyses, initial analyses were performed to identify confounders and effect modifiers by sequentially adding individual socio-demographic characteristics to the baseline model with the change score of each determinant as the dependent variable and ‘condition’ (intervention or control group) as independent variable. The following student-level characteristics were included: gender (boy and girl), education level (vocational level and high school/university level), and grade (first and second). The school location serves as school-level characteristic, determined by the population figures of the municipality where the schools are situated. Based on this criterion, they classified into two categories: (medium) large cities (>40,000 inhabitants) or town/small cities (≤ 40,000 inhabitants).^(26)^ Gender, education level, grade, and school location were found to be significant confounders for most determinants and behaviours. None of the characteristics were identified as significant effect modifiers, indicating that the effect of the programme did not differ for the determinants among subgroups defined by the measured characteristics. Subsequently, multivariate multilevel linear regression analyses were conducted, incorporating the change scores as dependent variables and including condition, grade, gender, education level, and school location as independent variables. Afterwards, the results of students who completed all (six) programme lessons were compared to those who completed only some. Results with a P-value <0.05 were interpreted as statistically significant.

## Results

### Characteristics of the study sample

The study sample included 611 students whose data were included in the analyses. The intervention group consisted of 364 respondents from eleven schools, while the control group consisted of 247 respondents from seven schools. The students had an average age of 12 ± 0.74 years, and the study sample displays a more or less balanced distribution across gender and grade (Table 2). The age of students and the percentage of second-year students in the intervention group were significantly lower compared to the control group (both P < 0.01). Furthermore, the intervention group had a significantly higher proportion of students at pre-vocational level (P < 0.01) and a higher number of students attending schools located in town/small cities compared to the control group (P < 0.01).

**Table 2.** Characteristics of the study sample (N = 611)

		Intervention group (n = 364)		Control group (n = 247)	
		Number	%	Number	%
Gender	*Boy*	178	49%	139	56%
	*Girl*	182	50%	106	43%
	*Other*	4	1%	2	1%
Age (mean ± SD)^*^		12.4 ± 0.74		12.9 ± 0.91	
Education level^*^	*Pre-vocational (general) secondary education*	218	60%	111	45%
	*Senior general secondary and pre-university education*	146	40%	136	55%
Grade^*^	*First*	218	60%	78	32%
	*Second*	146	40%	169	68%
School location*	*(Medium) large city*	151	42%	173	70%
	*Town/small city*	213	59%	74	30%

* Significant difference between the intervention group and control group, P < 0.01.

### Appreciation of the programme

According to the responses from the students in the intervention group, the overall rating for the programme was 6.9 ± 1.7 on a scale of 1–10. On average, students gave neutral to positive scores for the lessons and components of the programme (3.4 ± 1.0). The questions and videos (3.3 ± 1.0) were rated slightly more positive on a scale of 1–5 than the lessons (3.1 ± 0.7) and assignments (3.2 ± 0.8).

### Effect on behavioural determinants and behaviour

#### Capacity

In the pre-test, the students answered on average 43% of the knowledge questions correctly (Table 3). Most students (57%) answered the question regarding which product contains most dietary fibres correctly, while fewest students (29%) answered the question about the best choice of meat considering the environment correctly. Results showed a significant increase in knowledge between the pre-test and post-test in both the intervention and control group (both P < 0.01). The increase in knowledge among students in the intervention group (3.8 ± 1.6 vs. 4.3 ± 1.5) was not significantly higher than that among students in the control group (3.8 ± 1.5 vs. 4.3 ± 1.6) (P = 0.14).

**Table 3.** The average scores, the average change in scores of the intervention and control groups, and the results of the multilevel regression analyses

		Mean scores				Change scores		Difference in change	
		Pre-test		Post-test		Post-test – pre-test		Post-test – pre-test	
		Mean	(SD)	Mean	(SD)	Mean	(SD)	β	(SD)
**Capacity**									
*Knowledge*	Intervention group	3.81	(1.60)	4.34	(1.54)	0.35	(1.78)	0.00	(0.16)
	Control group	3.79	(1.47)	4.37	(1.60)	0.32	(1.67)		
*Self-efficacy*	Intervention group	3.34	(0.64)	3.63	(0.64)	0.29	(0.67)	**0.20**	**(0.07)** ^**^
	Control group	3.45	(0.74)	3.51	(0.75)	0.05	(0.80)		
**Opportunity**									
*Subjective norm*	Intervention group	5.05	(1.25)	5.07	(1.22)	0.02	(1.46)	0.10	(0.13)
	Control group	4.85	(1.23)	4.89	(1.34)	0.04	(1.33)		
**Motivation**									
*Attitude*	Intervention group	5.29	(1.09)	5.32	(1.06)	0.03	(1.09)		
								**0.29**	**(0.10)** ^**^
	Control group	5.32	(1.12)	5.21	(1.14)	–0.10	(1.09)		
*Intention*	Intervention group	3.30	(0.76)	3.34	(0.77)	0.04	(0.84)	0.11	(0.07)
	Control group	3.25	(0.76)	3.21	(0.78)	–0.05	(0.73)		
**Behaviour** ^a^									
*Fruit*	Intervention group	5	(4)	5	(4)	0.13	(1.82)	0.31	(0.17)
	Control group	5	(4)	5	(4)	–0.15	(1.70)		
*Vegetables*	Intervention group	6	(2)	6	(2)	0.08	(1.52)	0.08	(0.13)
	Control group	7	(2)	6	(2)	–0.11	(1.25)		
*Sugary drinks*	Intervention group	3	(4)	3	(3)	–0.48	(2.10)	**–0.37**	**(0.19)** ^*^
	Control group	3	(5)	3	(3)	–0.15	(2.06)		
*Whole grain bread*	Intervention group	2	(2)	2	(2)	–0.13	(1.19)	0.01	(0.11)
	Control group	2.5	(2)	2	(2)	–0.13	(1.20)		
*Sweet and savoury snacks*	Intervention group	4	(1)	3	(1)	–0.21	(1.14)	**–0.29**	**(0.10)** ^*^
	Control group	3	(1)	3	(1)	–0.01	(1.05)		
*Meat*	Intervention group	5	(3)	5	(4)	–0.33	(1.87)	**–0.34**	**(0.16)** ^*^
	Control group	5	(4)	5	(4)	0.02	(1.66)		

The multilevel analyses are corrected for grade, gender, education level, and school location of the students.

a Data were not normally distributed, thus the median + interquartile range (IQR) is presented. The change scores were normally distributed.

* P < 0.05.

** P < 0.01.

Intervention group: n = 347–364; control group: n = 231–247.

Regarding self-efficacy, students rated themselves slightly positive during the pre-test (3.4 ± 0.7 on a scale of 1–5). Only the intervention group showed a significant increase in self-efficacy (P < 0.01), and this increase was significantly higher than in the control group (P < 0.01).

#### Opportunity

During the pre-test, students had a positive average score for subjective norm (5.30 ± 1.24 on a scale of 1–7). There was no significant difference observed between the pre-test and post-test in both the intervention and control group and in the change between the intervention and control group (P = 0.44).

#### Motivation

During the pre-test, students had on average a positive attitude towards healthy, safe, and sustainable food choices (5.30 ± 1.10 on a scale of 1–7). Although there was no significant difference between the pre-test and post-test scores in both the intervention and control group, the change in the direction of a more positive attitude among students in the intervention group was significantly higher than in the control group (P < 0.01).

Regarding intention, the students had a slightly positive average score in the pre-test (3.28 ± 0.76 on scale of 1–5). Students scored their intention to eat less meat neutrally to slightly negatively (2.47 ± 1.24), while the intention to drink more water scored the highest (3.92 ± 0.98) of all intention items. There was a significant decrease in the intention to drink more water observed between the pre-test and post-test in the control group (P < 0.01), while the intention to drink more water did not change in the intervention group (P = 0.52), resulting in a significant positive difference in the intervention group compared to the control group (P = 0.04). However, there was no significant difference in the average intention to change other behaviours (P = 0.16).

#### Behaviour

During the pre-test, students most often reported eating fruit and meat five days a week (both 5 ± 4 (median and IQR)) and vegetables six days a week (6 ± 2). Students most often consumed sugar-sweetened beverages three days a week (3 ± 4). Most students reported consuming sweet or savoury snacks almost every day of the week (3 ± 2) and whole grain bread was most usually chosen (3 ± 1). In the intervention group, significant positive differences were observed on various behaviours compared to the control group, including consuming fewer sugar-sweetened beverages (P < 0.05), eating fewer sweet and savoury snacks (P < 0.01), and consuming less meat (P = 0.03).

When comparing only the students in the intervention group who completed all six lessons (n = 196) with the control group, the same results were observed for all outcome measures as when including all students from the intervention group (n = 364) in the analyses.

## Discussion

The results of the study indicate that students who participated in the ‘Weet wat je eet’ programme showed significant greater improvements in various behavioural determinants and behaviours related to healthy, safe, and sustainable nutrition compared to the control group. Specifically, the intervention group demonstrated a significant higher increase in self-efficacy, attitude, and intention to drink water, as well as in reducing consumption of sugary drinks, snacks, and meat than the control group. Both the intervention and control group showed increased knowledge scores at the post-test compared to the pre-test, but the intervention group did not show a statistically significant higher increase than the control group. There were no significant changes observed in subjective norms, intention, and behaviour related to consuming fruits, vegetables, and whole-grain bread between the intervention and control group. Although some positive outcomes were observed, other outcomes thus did not show significant changes. This mixed pattern of results suggests that the intervention may have limited overall efficacy and highlights the complexity of evaluating the effectiveness of educational interventions.

### Reflection on the results

The ‘Weet wat je eet’ programme aims to empower students to make healthy, safe, and sustainable food choices and foster a positive attitude towards this. The objectives of the programme have been partially achieved, as positive effects have been observed in terms of motivation (attitude and intention to drink more water) and partially on capacity (self-efficacy and knowlegde) and changes in eating behaviour aligned with the food-based dietary guidelines of the Dutch Wheel of Five (Schijf van Vijf) such as reducing consumption of sugary drinks, snacks, and meat.^(27)^ However, there is no observed effect on opportunity (subjective norm).

It is worth noting that this study did not reveal a significant effect on knowledge between the intervention and control group. This result was surprising given that schools are generally considered environments where knowledge is typically imparted, and previous nutrition education programmes have demonstrated increases in knowledge. This raises questions about the potential role of the assessment questionnaires themselves. It is possible that the act of completing these questionnaires increased awareness and prompted behavioural changes independent of the intervention content. Future studies should consider including additional control measures to isolate the effects of the intervention from the assessment process. Other Dutch programmes such as ‘Taste Lessons’^(14,20)^ and foreign programmes described in the systematic reviews by Murimi *et al.* and Shepherd *et al.* have reported a positive effect on knowledge.^(18,28)^ A possible explanation is that there is already increasing attention to healthy and sustainable food in secondary education in the Netherlands. It is plausible that the heightened focus on nutrition and lifestyle in recent times, as well as the inclusion of related topics in other subjects such as biology lessons, have contributed to the overall increase in knowledge levels observed in both groups. Therefore, the content of this programme may perhaps not have provided sufficiently distinct knowledge compared to what students already receive through other methods.

Literature shows that effective nutrition education should not solely focus on knowledge transfer but also focus on teaching skills, changing behaviour, and applying information in practice.^(29,30)^ In this study, although no significant effect on knowledge was found, positive effects were observed on students’ self-efficacy and motivation towards favourable eating behaviour. These findings align with the systematic review by Shepherd *et al.*, which reported that six out of seven interventions targeting attitudes towards healthy eating were effective.^(28)^

It is well established that adolescence is a crucial period in the development of nutrition behaviour among young people. This period is marked by increased independence, as young people start making their own food choices and are influenced by their friends and peers.^(31)^

According to Bruening *et al.*, large-scale research illustrates that eating patterns among adolescent friendship groups show similar eating patterns.^(32)^ This phenomenon may stem from young consumers’ desire to conform to social norms by mirroring their peer group’s eating behaviours. In the present study, no effect was observed regarding the opinions of friends of the students, which is in line with the lack of substantial change in subjective norm in other studies on nutrition education programmes fostering healthy eating among adolescents.^(33,34)^ Further research in this area could delve into exploring how to change the social and physical environment on food choices among young people.

A significant positive effect on the intention to drink water was found, which seems to be explained by a decrease in intention among the control group rather than an increase in the intervention group. No significant effect was found on other items of intention, which is in line with the results of another study on the effect of nutrition education on healthy eating behaviour intention.^(33)^ Despite the lack of effect on intention, which is seen as the most prominent predictor of healthy eating behaviour in adolescents,^(35)^ significant changes were observed in certain behaviours among students in the intervention group. Specifically, students in the intervention group reported a significant positive change in the consumption of sugary drinks, snacking, and meat consumption. No significant changes were found in the consumption of fruits, vegetables, and whole grain bread consumption. Research on another Dutch nutrition education programme ‘Krachtvoer’ (‘Power Food’), specifically targeting vocational education students, showed a significant increase in the consumption of vegetables and fruits, while no difference was observed in the consumption of fruit juices and high-fat snacks.^(36)^ This appears to be almost the opposite of the current findings. Studies conducted in Europe examining the effects of nutrition education programmes have reported mixed results in terms of vegetable and fruit consumption.^(37,38)^ According to a systematic review by Vézina-Im and colleagues focussing on soda consumption, which included 36 interventions, 65% of the education-based interventions led to a significant reduction in soda consumption among students.^(39)^ The results from previous studies, therefore, appear to be inconsistent. The effects on snacking behaviour and meat consumption in the current study have not been demonstrated before, to the best of our knowledge. This suggests the need for further research to explore the effect of nutrition education interventions on eating behaviour of adolescents.

### Limitations

To ensure a proper interpretation of the results, it is important to consider several limitations. The data obtained in this study relied on self-reported information assessed through (online) questionnaires, which may potentially be subject to social desirability biases. Additionally, it is unclear whether the students were capable of accurately translating their opinions into the appropriate questionnaire responses, and whether the questionnaire used the most suitable questions and response categories. However, efforts were made to address these concerns during questionnaire development and testing, such as controlling the terminology at a suitable level and involving students and a teacher in the pilot testing. Although the questionnaire was carefully developed and pre-tested, not all students completed it in its entirety. Analysis revealed a higher number (4%) of unanswered questions on the last pages of the questionnaire.

The decision was made to include in the analysis only those students who used the same first name in both the pre-test and post-test and were in the same class. As a consequence, this led to the exclusion of a substantial portion (43%) of responses from the pre-test in the analysis. Possible reason for the drop-out include the effort to minimise the collection of personal data to protect student privacy, which may have led to a lack of follow-up. A baseline comparison on characteristics and the outcome measures between completers and non-completers showed no significant differences, suggesting that the drop-out did not introduce bias in the sample. It is conceivable that this exclusion resulted in a selective group of students who may have filled out the questionnaire twice, or who might have been easily distracted, had difficulty completing questionnaires, or lacked the motivation to complete the questionnaire seriously. This may have led to the absence or underrepresentation of a selective group of students in the results, potentially affecting the generalisability of the findings. Since the dropout of participants occurred in both the intervention and control groups, it is not expected to have influenced the results. However, the dropout of participants may have resulted in lower statistical power, potentially leading to the failure to detect true effects.

In this study, multiple statistical tests were conducted to examine various outcomes related to the intervention. This increases the risk of Type I errors. To address this, the use of Bonferroni correction and other statistical adjustments was considered. However, it is also recognised that such adjustments can elevate the risk of Type II errors, potentially obscuring meaningful findings. Therefore, a balanced approach was taken in interpreting the results, considering both statistical significance and practical relevance.

A quasi-experimental research design was used. The inclusion of a control group and the use of a pre-test provide some confidence in attributing the observed results to the programme. However, the allocation of schools to the intervention or control group was not random but based on their voluntary participation in implementing the ‘Weet wat je eet’ programme. In this study, the intervention and control group differed in terms of sociodemographic characteristics. In the Netherlands, the distribution of students in the first and second grade is approximately fifty-fifty, with slightly more boys than girls in the lower years.^(26)^ The distribution of grade and gender in both the intervention and control groups in the current study deviated from this general distribution. The analyses on change scores between the intervention and control group were adjusted for these differences, including grade, gender, education level, and school location. Nevertheless, differences between the groups may have influenced the results. For future research, it is recommended to ensure a comparable distribution of characteristics to enhance the reliability and generalisability of the results.

### Conclusions

The findings of this study indicate that the ‘Weet wat je eet’ school-based nutrition education programme is effective in enhancing self-efficacy and attitude towards healthy, safe, and sustainable nutrition, as well as the intention to increase water consumption and certain self-reported behaviours among secondary school students aged 12–15 years. No significant effects were observed for knowledge, subjective norm, intention, and fruit, vegetable, and whole grain bread consumption. Although there was an increase in students’ nutrition knowledge, it did not show a statistically significant greater improvement in the intervention group compared to students who did not receive the programme. Further research is necessary to examine the long-term sustainability of these positive changes over an extended period.
